# Associations between Physical Activity and Health-Related Quality of Life among Community-Dwelling Older Adults: A Cross-Sectional Study in Urban Greece

**DOI:** 10.3390/geriatrics8030061

**Published:** 2023-06-01

**Authors:** Anna Psarrou, Theodoula Adamakidou, Paraskevi Apostolara, Alexandra Koreli, Marianna Drakopoulou, Sotirios Plakas, Dimos Mastrogiannis, Alexandra Mantoudi, Stelios Parissopoulos, Afroditi Zartaloudi, Marianna Mantzorou

**Affiliations:** MSc Program in Community and Public Health Nursing, Nursing Department, School of Health and Care Sciences, University of West Attica, 12243 Athens, Greece; annapsarrou6@gmail.com (A.P.); thadam@uniwa.gr (T.A.); vapostolara@uniwa.gr (P.A.); akoreli@uniwa.gr (A.K.); mdrakopoulou@uniwa.gr (M.D.); skplakas@uniwa.gr (S.P.); dmastrogiannis@uth.gr (D.M.); amantoudi@uniwa.gr (A.M.); spariss@uniwa.gr (S.P.); azarta@uniwa.gr (A.Z.)

**Keywords:** aging, COVID-19 pandemic, older adults, physical activity, quality of life

## Abstract

Physical activity is an important factor in achieving healthy aging, offering older persons multiple benefits in terms of maintaining and improving their health and wellbeing. The aim of this study was to investigate the effect of physical activity on the quality of life of older adults. A cross-sectional study was conducted from February to May 2022, using the Short-Form Health Survey (SF-36) and the International Physical Activity Questionnaire (IPAQ). A total of 124 people aged 65 and over participated in the survey. The average age of the participants was 71.6 years, and 62.1% were women. Participants showed a moderate quality of life with regard to the physical health dimension (mean score 52.4) and a higher quality of life with regard to the mental health dimension (mean score 63.1) compared to the expected values of the population. Low levels of physical activity were recorded among older adults, reaching a rate of 83.9%. A moderate or high level of physical activity has been found to contribute to a better physical functioning (*p* = 0.03), vitality (*p* = 0.02) and general health (*p* = 0.01). Finally, comorbidity had a negative impact on physical activity (*p* = 0.03) and quality of life regarding mental and physical health in older adults. The study showed very low levels of physical activity in older Greek adults. The management of this problem, which was intensified during the COVID-19 pandemic, should be a high priority in public health programs focusing on healthy aging, as physical activity affects and promotes many of the basic aspects of quality of life.

## 1. Introduction

Aging is a complex, normal and inevitable process in human life, characterized by biological, psychological and social changes which occur in each person at different rates due to various influencing factors [1]. The World Health Organization (WHO) has declared that every person—in every country in the world—should be given the opportunity to live a long life in good health and full participation in society. Healthy aging has been the focus of WHO’s work between 2015 and 2030 and has been defined as “the process of developing and maintaining the functional ability that enables well-being in older age” [2].

In an effort to support older people to be active and obtain a satisfactory level of good health and mood, many interventions have been sought. An important factor in achieving healthy aging is physical activity (PA), which has been positively associated with a number of benefits for maintaining and promoting the health-related quality of life (HRQoL) of the older population [3,4,5,6,7,8,9].

Despite the sudden demographic, epidemiological and anthropological changes triggered by the aging process, physical activity contributes to healthy and quality life years. The American College of Sports Medicine in cooperation with the American Medical Association have established the «Exercise is Medicine» health initiative, which aims to promote the value of physical activity and highlight its multiple benefits. These benefits are more pronounced in people who give up inactivity and a sedentary lifestyle and adopt a more active one [10].

Physical activity is a significant strategy for achieving healthy aging, reducing early mortality by 20–30%, while it beneficially impacts wellbeing (mental, emotional and physical) [5]. Hamer et al., (2014) claim that even a delayed start in physical activity results in remarkable health benefits [11]. According to a study conducted by Balboa-Castillo et al. in 2011 on Spanish people over 60, even light, leisure physical activity such as walking has a positive impact on older adults’ physical, mental and social sphere of life [3]. Other studies similarly claim that light or moderate activities such as walking, gardening, swimming, cycling and dancing benefit the physical, emotional and social wellbeing of older persons [4,8].

Additionally, a study of older persons in Seoul, Korea, conducted by Park et al., (2020) determined that activities boosting physical strength, during which low- or medium-level energy was expended, such as gardening, were beneficial to physical and cognitive health, the psychological state and the prevention of depression [12]. Older persons in Australia who spent time tending to their gardens, and therefore had the opportunity for mental and physical activity, developed, as a result, a positive attitude towards aging and by extension had a better quality of life [13]. Finally, the use of bicycles by older adults brings spectacular results as it increases physical strength, stimulates cognitive function, promotes wellness and enhances their mental health [14].

Participation in primary health and social care centers for older people can promote network growth and access to social resources, increase self-esteem, reduce loneliness and increase physical activity [15]. Participation in community centers where older persons can attend recreational activities and physical exercise programs such as aerobic exercise or traditional dance programs have been found to promote physical health and social interaction and positively affect their mental wellbeing, thus improving their quality of life [16,17]. Older persons who were deprived of the beneficial effects of activities in community centers during the COVID-19 pandemic presented an increased level of loneliness and depression [18]. Thus, innovative exercise programs are suggested and applied, making use of applications and the Internet of Things (IoT) so as to promote older persons’ quality of life. Wearable biomedical sensors can help monitor the older person’s mobility and physiological parameters. A smart home system may include smart fitness equipment in order to help older people perform rehabilitation and exercise programs [19].

Published findings continuously confirm that higher physical activity in the work and home environment or during leisure time can promote people’s quality of life, regardless of existing ailments [20]. Badicu (2018) reports a very positive correlation between the physical activity and quality of life of an adult, regardless of the type of exercise [21]. Subramaniam et al., (2019), while studying the relation between physical activity and quality of life in people with comorbidities, found a strong positive correlation and noted that the lower the levels of physical activity, the poorer the HRQoL [22].

The literature includes little evidence regarding older adults and reveals heterogeneous results regarding physical activity and HRQoL in older people, partly because of the different research designs and different research tools used in order to measure physical activity and HRQoL. In addition, many of the studies concerned older people living in nursing homes instead of community dwellers [23,24]. Studies conducted in Greece investigating the relationship between certain types of physical activity (i.e., traditional dancing) and quality of life and how these affect the life of older people are few in number, making it difficult to extract meaningful and valid conclusions [16,17,25,26]. Moreover, they were conducted before the COVID-19 pandemic, which affected older persons’ lives and created another “pandemic” of inactivity and sedentary lifestyle [27]. The current study comes to fill in the gap in the existing literature by examining the relationship between PA and HRQoL using standard measures of all the dimensions of HRQoL.

## 2. Objective

The aim of this study was to explore the relationship between physical activity and quality of life in community-dwelling older adults in Greece during the third year of the pandemic so as to utilize the results towards promoting healthy and active aging.

## 3. Materials and Methods

### 3.1. Study Materials

The current study is an exploratory cross-sectional study carried out through self-report questionnaires. The sample of the study was a convenience sample of 124 participants > 65 years who could speak, read and write in Greek. Older persons with cognitive impairment (according to their medical file) or serious mobility problems who were not able to be physically active were excluded from the study. The study took place in walk-in day centers for older people and primary health care and social care services in the south and central sector of the Athens region during the period February–May 2022. Although community centers for older people remained closed for quite a long period during the pandemic in Greece, during the study period, vaccinated persons only, wearing face masks, could visit public services. Walk-in day centers were accepting only small numbers of older people who had been vaccinated. No recreational activities such as aerobic exercise or traditional dance were taking place during that period, just medical prescriptions and physiotherapy sessions. Wearing face masks was still obligatory when walking in open public places, which was also an inhibiting factor for physical activity outdoors.

### 3.2. Ethical Approval

Written permission for the study was granted by the health regions and the corresponding municipality services. Permission was also granted by the Ethics Committee of the University of West Attica (protocol number 17349; date: 23 February 2022).

Participants were informed about the scope and the possible benefits of the study. They were assured of the confidentiality of personal data, and that they were free to withdraw at any stage. Finally, they signed an informed consent form.

### 3.3. Data Collection

The main researcher recruited participants who were visiting the primary health and social care services and examined the eligibility criteria in collaboration with local healthcare professionals. After participants signed the consent form, the first author, who was the main researcher, personally distributed the questionnaires in a quiet and private area of the facilities. Out of the 130 participants recruited, 124 completed the questionnaires, yielding a response rate of 95%. According to the G-power program, the number of 130 participants was found to be sufficient for a correlation study in which a multivariate linear regression analysis will be performed with a power of 95%, an error level of α = 0.05, an effect size of at least 0.16 and a total number of controlled predictors of 5. A post hoc analysis revealed that the actual sample size provided a level of power of 95% to detect an effect size of at least 0.167, a value that is almost identical to our initially intended one.

### 3.4. Instruments

A sociodemographic and clinical characteristics questionnaire as well as the following scales were used.

#### 3.4.1. Short-Form Health Survey (SF-36)

The SF-36 is a generic research tool used for the assessment of physical, cognitive and mental health of populations and patient groups (Medical Outcomes Trust, Boston, MA, USA) [28,29]. License for use was granted by the RAND Health Care company without any need for written permission. SF-36 has been validated in the Greek population [30,31] and it has been found to be a reliable tool with Cronbach’s α coefficient ranging from 0.79 to 0.95. It includes 36 questions, which make up 8 subscales, with 2 to 10 questions each: The individual subscales/dimensions of physical health are: (1) Physical Functioning (PF), (2) Role Functioning/Physical (RP) (Role limitations due to physical health problems), (3) Bodily Pain (BP) and (4) General Health (GH), while mental health includes the additional subscales/dimensions: (5) Vitality (VT), (6) Social Functioning (SF), (7) Role Functioning/Emotional (RE) (role limitation due to emotional problems) and (8) Mental Health (MH). The answer choices range from 2 to 5 ratings. Two summary component scales, the Physical Component Summary and the Mental Component Summary, derive from the 8 subscales. The score of the SF-36 subscales and components ranges from 0 to 100, while the mean value is 50 [32]. Expected score values for the mental health component vary between 17 to 62, while for the physical health component they vary from 20 to 58 [33]. The mean value of the Greek general population is 51.99 for PCS and 49,73 for MCS. Lower values are associated with worse mental and physical quality of life, respectively, and the higher the score, the better the health [34].

#### 3.4.2. International Physical Activity Questionnaire (IPAQ)

The International Physical Activity Questionnaire (IPAQ), developed by Craig et al., (2003), was validated on adults across twelve countries (Spearman’s ρ averaged around 0.8 and the criterion validity median ρ was about 0.30) [35]. The Greek version presented good to high reliability properties (intra-class correlation coefficients (ICCs) of 0.69 to 0.93) [36]. The IPAQ contains questions related to physical activity in both leisure time and at the workplace, at home and during participants’ daily commutes. Participants report the number of days (frequency) and the number of minutes per day (duration) that they spend in all kinds of vigorous, moderate and walking physical activities during the past week. In addition, a seventh question records the time that subjects spend sitting during an average weekday. The total number of questions is 7 and the grading classifies the level of physical activity into (1) low, (2) moderate and (3) high [36]. Participants who do not meet the criteria of level 2 and 3 are characterized as inactive [37]. Although IPAQ was originally constructed for use in adults 18–65 years old, it has also been used for the measurement of physical activity and sedentary behavior in older adults. Research has shown that IPAQ is a valid tool for the measurement of physical activity and sedentary behavior in older adults in South Africa, showing good reliability and criterion validity (*r* = 0.46 to 0.77) [38]; in Belgium (moderate validity, *r*  =  0.33–0.40) [39]; Japan (adequate validity, *r*  =  0.42–0.53) [40]; Hong Kong (acceptable reliability and validity, *r*  =  0.47) [41] and the United Kingdom (moderate/acceptable levels of validity (*r*  =  0.430–0.557) for moderate/vigorous physical activity (PA) [42]. It has also been tested by qualitative methodologies, showing its usefulness in older populations provided that clear explanations and directions are given to older participants [43]. The IPAQ is an open access questionnaire and the team that developed it notes that no permission is required in order to use it.

### 3.5. Data Analysis

Categorical variables are presented with absolute (*n*) and relative (%) values, while quantitative variables are presented with standard deviations, means, medians and range of values. The Kolmogorov–Smirnov test and normality diagrams were used to examine the normal distribution of quantitative variables. Statistical tests such as the x^2^ test, *t*-test and Pearson’s and Spearman’s correlation coefficients were employed for data analysis. Multivariate linear regression was performed to identify variables associated with quality of life, employing the backward stepwise entry method. Beta coefficients, 95% confidence intervals and *p* values are presented. Multivariate logistic regression was performed to identify variables associated with physical activity. Odds ratios with corresponding 95% confidence intervals and *p* values are presented. Statistical significance level was set at 0.05. Data analysis was performed with the Statistical Package for Social Sciences (SPSS) v.21.

## 4. Results

### Demographic and Clinical Characteristics

**Figure 1:**
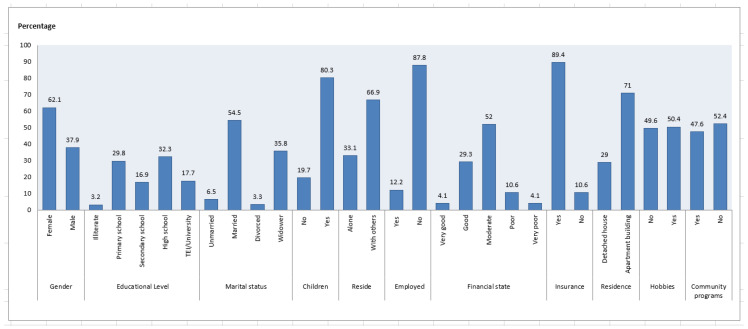
Sociodemographic characteristics of the participants.

The sample under study consisted of 124 older adults whose sociodemographic and clinical profile is presented in Figure 1. Their average age was 71.6 years, and 62.1% were women. Of the participants, 32.3% were high school (lyceum) graduates, while 17.7% also had a higher education degree. The majority of participants (54.5%) were married, had children (80.3%) and were living with other people (66.9%). Most of the participants were retired (65%), had a health insurance (89.4%) and regarded their financial state as moderate (52%). Half of them had a hobby (50.4%) and participated in community programs for older people (47.6%) (Figure 1).

**Figure 2:**
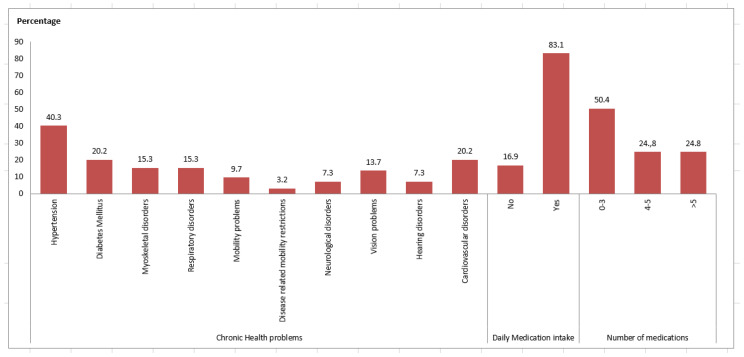
Clinical characteristics of the participants.

The clinical characteristics of the older persons are presented in Figure 2. The majority of participants (75.2%) had a chronic disease, most commonly, hypertension, diabetes, cardiovascular disease and vision problems. Most of them (83.1%) were taking medication for their chronic disease (Figure 2).

The level of physical activity of the older adults is presented in Table 1. Of the participants, 83% presented a low level of physical activity, 8.9% a moderate level and 7.3% a high level. The scale presents a good level of split-half reliability (0.889).

The descriptive results of the SF-36 subscales as well as each subscale’s Cronbach’s α value are presented in Table 2. Cronbach’s α values ranged between 0.76 and 0.94, which indicates a very good reliability of the SF-36. Higher scores of the SF-36 indicate a better quality of life.

Scores on the Mental and Physical Component Summary dimensions were 52.4 for PCS (expected mean value of PCS: 51.99 in the Greek general population) and 63.1 for MCS (expected mean value of MCS: 49.73 in the Greek general population) (Table 2).

Comparisons between participants’ low and moderate/high activity levels (IPAQ) and SF-36 subscales are presented in Table 3. Participants with a moderate or high level of physical activity had higher scores with regard to Physical Functioning (*p* = 0.03), Physical Role (0.03), Vitality (*p* = 0.02) and General Health (*p* = 0.01) compared to participants with a low level of physical activity (Table 3).

According to the results of the multivariate linear regression analysis, with general health as the dependent variable, it emerged that older persons with a higher educational level (*p* = 0.048), those who had hobbies (*p* < 0.001) and those who had children (*p* < 0.039) had better general health. In contrast, the older adults with more comorbidities (*p* = 0.003) had worse general health (Table 4).

The MCS dimension was found to be positively associated with higher educational level (*p* = 0.001) and negatively associated with comorbidity (*p* = 0.013) (Table 4). No statistically significant results were found in the multivariate linear regression analysis with the PCS as the dependent variable.

According to the results of the multivariate linear regression analysis, for Physical Functioning, it appears that high school graduates and married people had better physical functioning (*p* = 0.004 and *p* = 0.0019, respectively), while the persons with more comorbidities had worse physical functioning (*p* < 0.001) (Table 4).

An important finding of the study is the fact that comorbidity was negatively associated with Physical Functioning (*p* < 0.001), Physical Role (*p* < 0.001), MCS scale (*p* = 0.013), Body Pain (*p* = 0.002), General Health (*p* = 0.003), Vitality (*p* < 0.0010, Social Functioning (*p* = 0.001), Emotional Functioning (*p* < 0.001) and Mental Health (*p* = 0.005).

Finally, with regard to the level of physical activity, according to the results of the multivariate logistic regression, it appears that the older persons who had hobbies had a higher level of physical activity. Older adults with hobbies were 3.5 times more likely to have a moderate/high level of physical activity than older adults without hobbies, and older adults with fewer comorbidities had a higher level of physical activity. The odds ratio of a high level of physical activity decreased by 0.6 for each additional disease from which the older persons suffered (Table 5).

## 5. Discussion

The present study was undertaken to investigate the relationship between physical activity and quality of life in community-dwelling older adults over 65 years old.

Following the analysis of quality of life data derived from the SF-36 scale, it was found that the mean value of the summary mental health dimension was 63.1 (mean value of MCS: 49.73 in the Greek general population), while the mean value of the summary physical health dimension was 52.4 (mean value of PCS: 51.99 in the Greek general population). This shows that older adults had better quality of life with regard to mental health than physical health. The norms suggested by Papa et al., (2005) [31] and Karapanou et al., (2012) [44] that were used in the present study derived from a validation and norming study in a large sample of the urban Greek population which revealed similar norms with the reference study of Ware, thus allowing comparisons with other countries [31]. The lower level of physical compared to mental health is a finding consistent with studies on older populations. Sun et al., (2015) found similar values for mental and physical health (58.9 and 53.7, respectively) [45]. This finding is also similar to the results of Attafuah et al., (2022), who found a poor level of physical health and a moderate level of mental health [46].

The level of quality of life with regard to the mental health of the participants in our study was found to be higher than that of the general Greek population (mean value of MCS: 63.1 compared to MCS: 49.73 in the Greek general population). This finding is in accordance with the findings of other studies which showed that the levels of anxiety and depression of older people during the COVID-19 pandemic were lower than those of younger adults [47,48,49]. Although the levels of depression and anxiety were higher than those in the pre-COVID era [50,51], they were lower than those of younger adults, probably because younger people had to face unemployment, childcare and distance learning [50]. A study in Singapore showed that older adults were more psychosocially adaptable, which may also explain the better mental health of older adults [49].

The better mental health of older people in our study compared to the expected score values in the US general population (expected scores for mental health component are 17 to 62) may be related to stronger family ties, which reduce feelings of loneliness and isolation resulting in fewer depressive symptoms even in the last period of the pandemic. In fact, at the time the study was conducted, a very large part of the older population had been vaccinated and the number of deaths had significantly decreased, which had reduced the negative effects on the mental health of older people during that period [18]. Quality of life in relation to physical health, on the contrary, seems to have remained at moderate levels, obviously affected by the restrictive measures and the effects of the pandemic, since, as already mentioned, the walk-in centers for the elderly operated with restrictions and activities such as creative activities, exercise and recreation were not carried out.

An important finding in the current study is the very high percentage (83.9%) of inactive older people, which poses serious risks to their health, compared to only 7.3% who presented a high activity level. This finding is in accordance with another study using IPAQ on Greek older adults on hemodialysis, in the third year of the pandemic, which found that the majority of older persons presented inactivity (69.7%). Persons exhibiting moderate and high activity represented 28.8% and 1.5% of the sample, respectively [52]. Another study conducted in Greece during the COVID-19 pandemic, also using IPAQ, highlighted the insufficient level of older adults’ physical activity (low 44%, moderate 42.6 and high activity 13.4% [53]. All studies mentioned showed insufficient levels of physical activity among older adults compared to the suggested levels by WHO (2020) (150–300 min of moderate-intensity aerobic physical activity weekly) [54]. The level of physical activity of older adults decreased during the pandemic in Greece; research data collected before the pandemic present a higher level of regular activity, although still insufficient [55]. Other studies carried out on an international level before the pandemic also indicate that the vast majority of older people present a lack of daily exercise, resulting in comorbidities and the deterioration of their general health [56,57]. The inactivity pandemic, though, that followed the COVID-19 pandemic was a worldwide phenomenon which deteriorated the activity of older persons [27,58]. It is noteworthy that women in our study reported lower levels of physical activity than men, a finding, of course, open to interpretation; men usually have more free personal time at their disposal, contrary to women who, even at older ages, spend most of their time in household activities or looking after their children and grandchildren, showing the gender gap regarding different amounts of free personal time [55,59]. Similar conclusions have also been obtained in the research of Souza et al., (2015), where physical inactivity was found to be increased in older women compared to men [60].

In the present research, a moderate or high level of physical activity was found to contribute to a better physical functioning (*p* = 0.03), vitality (*p* = 0.02) and general health (*p* = 0.01) compared to a low level of physical activity. However, further analysis by multivariate linear regression has not found physical activity to be an independent predictor of quality of life. This is probably due to the particularly high percentage of older people with low levels of physical activity (83.9% low and 8.9% moderate), a result which was expected due to the ongoing effects of the pandemic during the period of the study (February–May 2022), where vaccinations had progressed and death rates had decreased, but the negative effects on the physical condition and psychology of the elderly were still intense. Additionally, the walk-in centers for older people did not carry out creative exercise and leisure activities, and the use of masks was still mandatory outdoors, where older people could exercise, both significant inhibiting factors for physical activity. Therefore, activity levels recorded during the present study were not sufficient to increase the quality of life of older persons in this time period. In line with our results, the study by Sepúlveda-Loyola et al., (2020) also determined the effects of the COVID-19 pandemic on the mental and physical health of the elderly. Increased physical inactivity and high levels of loneliness, depression, anxiety and poor sleep quality were observed [61]. Furthermore, Ghram et al., (2020) clarified that the isolation of the elderly during this period increased sedentary behavior and resulted in the presence of functional disorders, an increase in morbidity and the risk of falls [62]. Encouraging participation in physical activity addresses the health problems of the elderly related to physical inactivity by significantly improving physical fitness, cognitive performance and quality of life [63,64].

Another finding of the present study was that about half of the elderly (47.6%) participated in community programs or spent time partaking in a favorite hobby (50.4%), which simultaneously develops social functioning (*p* = 0.002). It has been reported that a wide range of activities, such as hobbies, traveling, going out and participating in group activities give older people joy and meaning in their lives, removing sadness and apathy [65]. In our study, it was found that the older persons who had included some favorite occupation in their daily life recorded satisfactory levels of physical activity, showing 3.5 times more frequent moderate- or high-level physical activity compared to older people who did not have hobbies. Participating in physical activity helps maintain and improve health, physical function and quality of life [16,17,66,67], while physical inactivity is considered one of the most powerful factors for the deterioration of physical and mental health in older people [68].

In the present study, a higher educational level was associated with better mental health (MCS), better physical functioning (PF) and better general health (GH). This could be attributed to the fact that older people with a satisfactory level of education acquire a higher level of health literacy and thus have the ability to deal with and manage issues related to their health better [69]. These findings are in line with the international literature, as evidence shows that people with a good educational level engage in healthy behaviors, which leads to improvement in their physical health [70]. In addition, educational level can positively contribute to the psychological state of older persons and their social relationships. In numerous studies, a higher educational level was associated with a better quality of life [71,72,73,74,75]. Through education, cognitive abilities are increased, unhealthy habits are prevented, diseases can be managed and the quality of life is holistically improved [76].

Another important factor that affects older persons’ perception of quality of life are comorbidities. A possible explanation could be the fact that chronic diseases and geriatric syndromes in older people contribute to their subjective perception of poor health status [77]. In the current study, the observed levels in mental health, physical, social and emotional role, vitality, general health and physical activity were all found to be affected by comorbidities. The negative impact of multiple and chronic diseases on various aspects related to the quality of life of older persons is evident in many studies [78,79,80,81,82].

Finally, the current study revealed a noticeable negative correlation between comorbidity and physical activity, a fact that can be justified since the progressive deterioration of bodily functions limits mobility and creates fragile health, and the pain that occurs causes psychological discomfort and a lack of desire to exercise. Specifically, older adults with fewer comorbidities had a higher level of physical activity. This finding contradicts the results of the research of Sadrollahi et al., (2016), where older people with chronic conditions had a higher level of physical activity in effort to change their attitude towards the way of dealing with their illness and life in general [83]. Research data show that during the pandemic, older adults with comorbidities decreased their physical activity levels and were more likely to be hospitalized [84,85].

### Study Limitations

The results of the current study are subject to some limitations, as the research was not conducted at a nationwide level, but at specific primary health and social care services in the prefecture of Attica, using convenience sampling, and as a result, their generalizability is reduced. At the time of the study, older people were still reluctant to visit primary health and social care services because there was still a fear of COVID-19, and convenient sampling was the most appropriate technique in order to recruit all available eligible persons.

Additionally, the physical activity and quality of life of older persons were only assessed using self-administered questionnaires. In addition, the mental health of participants was not assessed prior the questionnaires’ completion. Moreover, norms used to compare the findings of the current study with regard to the physical and mental health component were derived from a Greek general population instead of a geriatric population. In addition, it is worth considering that the research was carried out during a particularly difficult period where, due to the pandemic, physical activity had been significantly decreased and the physical and psychological condition of older people had been affected. Finally, the use of IPAQ, which is not a tool specifically designed for older adults, is a possible limitation of the study. It should be mentioned that older adults present more difficulties recalling physical-activity-related data, especially lower-intensity and unstructured physical activity, adding a bias in the results [86,87]. However, younger adults may have similar difficulties in recalling activities of low intensity and duration [43]. The main researcher who conducted the interviews was specially trained and used all recommended techniques to minimize error possibly deriving from the use of a non-geriatric tool in older adults (careful explanation of questions, repetition of intensity and time criteria and provision of examples) [43].

Despite any limitations and difficulties, the results obtained from similar research are considered particularly useful for understanding how physical activity is linked to quality of life and for conducting further research work aiming to develop sustainable interventions in public health.

## 6. Conclusions and Recommendations

Aging is an inevitable process of human nature; however, there are approaches that can promote healthy aging by slowing down the negative effects, postponing degeneration, preserving activeness and sociability and securing an independent life, with physical activity being one of them.

Focusing on the results of the present study, we conclude that physical activity influences and promotes many key aspects of quality of life. Physical activity seems to have an influence on physical functioning as well as a positive impact on general health and vitality of the participants.

The beneficial effect of physical activity on the overall health and quality of life renders it highly important as a public health strategy. The adoption of an active lifestyle must be part of the planning and promotion of healthy aging as a factor that provides independence, functionality, longevity and quality of life among older people.

The activation of institutions (family, city, community, associations and clubs), the design of successful interventions focusing on the increase in physical activity, the enhancement of health literacy of older adults on the benefits of physical activity and the encouragement and motivation for the improvement in physical fitness are useful strategies for the preservation of the physical and mental health of this population as well as their ability to experience healthy and active aging.

## Figures and Tables

**Table 1:** Level of physical activity of older adults.

Level	Ν	%
Low	104	83.9
Moderate	11	8.9
High	9	7.3

**Table 2:** Descriptive results of SF-36 subscales.

Subscales	Mean	Standard Deviation	Median	Lowest Value	Highest Value	Cronbach’s α
Physical Functioning (PF)	69.7	30.8	75	0	100	0.94
Role Functioning/Physical (RP)	64.1	28.1	69	0	100	0.76
Bodily Pain (BP)	58.1	25.4	62	0	100	0.73
General Health (GH)	56.2	19.8	57	5	97	0.86
Vitality (VT)	57.7	20.6	63	6	100	0.89
Social Functioning (SF)	67.8	26.6	68	12.5	100	0.84
Role Functioning/Emotional (RE)	68.2	28.2	75	0	100	0.81
Mental Health (MH)	59.2	22	58	5	100	0.86
Physical Component Summary (PCS)	52.4	14.7	55.3	14.3	62.8	0.88
Mental Component Summary (MCS)	63.1	16.7	63.8	23.3	97.2	0.91

**Table 3:** Comparisons between participants’ low and moderate/high activity levels (IPAQ) and SF-36 subscales.

Subscales	Physical Activity ^a^ (Low vs. Moderate/High) *p* Value
Physical Functioning (PF)	0.03
Role Functioning/Physical (RP)	0.03
Bodily Pain (BP)	0.1
General Health (GH)	0.01
Vitality (VT)	0.02
Social Functioning (SF)	0.1
Role Functioning/Emotional (RE)	0.1
Mental Health (MH)	0.1
Physical Component Summary (PCS)	0.6
Mental Component Summary (MCS)	0.6

^a^: *t*-test.

**Table 4:** Multivariate linear regression analyses’ results of SF-36 for the sample.

Independent Variable	B Coefficient	95% Confidence Interval of b	*p* Value	Collinearity Diagnostics	
				Tolerance	VIF
Mental Component Summary (MCS) as Dependent Variable R^2^ = 0.188					
High school compared to primary school/illiterate	13.9	5.6–22.2	0.001	0.88	1.13
Secondary school compared to primary school/illiterate	10.9	0.6–21.1	0.038	0.90	1.11
Comorbidity	−3.6	−6.4–−0.8	0.013	0.97	1.02
**Physical Functioning (PF)** **as Dependent Variable R^2^ = 0.238**					
High school compared to primary school/illiterate	13.6	2.3–24.9	0.004	0.83	1.21
Married	11.9	2.1–21.8	0.019	0.97	1.03
Comorbidity	−8.2	−12.1–−4.3	<0.001	0.88	1.14
**Role Functioning/Physical (RE)** **as Dependent Variable R^2^ = 0.30**					
Married	10.6	1.8–19.4	0.018	0.94	1.06
Comorbidity	−7.8	−11.1–−4.6	<0.001	0.97	1.03
Hobby	13.2	4.6–21.7	0.003	0.98	1.02
**Bodily Pain (BP)** **as Dependent Variable R^2^ = 0.17**					
Comorbidity	−5.5	−8.9–−2.1	0.002	0.88	1.14
**General Health (GH)** **as Dependent Variable R^2^ = 0.243**					
High school compared to primary school/illiterate	7.8	0.6–15.1	0.035	0.83	1.21
Higher education compared to primary school/illiterate	8.9	0.07–17.9	0.048	0.81	0.123
Hobby	1.4	5.1–17.6	<0.001	0.88	1.14
Comorbidity	−3.8	−6.3–−1.3	0.003	0.87	1.14
Number of children	3.0	0.2–5.9	0.039	0.99	1.01
**Vitality** **as Dependent Variable R^2^ = 0.20**					
Married	8.2	1.5–15	0.017	0.95	1.05
Comorbidity	−5.5	−8.1–−3.1	<0.001	0.97	1.02
**Social Functioning (SF)** **as Dependent Variable R^2^ = 0.19**					
Male	9.9	0.4–19.4	0.041	0.89	1.11
Participation in community programs	13.9	5.1–22.6	0.002	0.99	1.01
Comorbidity	−5.3	−9.1–−2.4	0.001	0.96	1.04
**Role Functioning/Emotional (RE)** **as Dependent Variable R^2^ = 0.21**					
Married	17.7	8.5–26.8	<0.001	0.99	1.00
Comorbidity	−7.2	−10.7–−3.8	<0.001	0.99	1.00
**Mental Health (MH)** **as Dependent Variable R^2^ = 0.146**					
Comorbidity	−4.1	−7.1–−1.3	0.005	1.00	1.00

**Table 5:** Multivariate logistic regression analysis of physical activity as dependent variable.

	Physical Activity as Dependent Variable R^2^ = 0.19		
	Odds Ratio	95% Confidence Interval	*p* Value
Hobby	3.5	1.1–11.1	0.03
Comorbidities	0.6	0.3–0.9	0.03

## Data Availability

Datasets used and/or analyzed during the current study are available on reasonable request from the corresponding author. The data are not publicly available due to privacy reasons.
